# Comparative Analysis of Physicochemical Composition and Antioxidant Properties of Three Edible Mushroom Varieties

**DOI:** 10.3390/molecules31172950

**Published:** 2026-08-23

**Authors:** Oluwaseun Peter Bamidele

**Affiliations:** Department of Food Science and Technology, University of Venda, Thohoyandou 0950, South Africa; oluwaseun.bamidele@univen.ac.za

**Keywords:** mushroom, phenolic compounds, antioxidant, mineral, water activity

## Abstract

This manuscript presents findings from the analysis of three species of Pleurotus mushrooms common in Ile-Ife, Nigeria, detailing evaluations of proximate composition, physicochemical properties, minerals, colour, phenols, and antioxidant capacity. Standard analytical procedures were used to analyse the mushroom flour samples. Moisture content was within the range of 9.56% (*P. florida*) and 11.01% (*P. pulmonarius*), indicative of good drying practices. Protein content among the three species was high, with that of *P. ostreatus* showing the highest value (26.12%), and all had low fat content (3.45–3.70%). The physicochemical analysis showed pH values between 6.12 and 6.31, indicating neutral pH, and perishability was indicated by water activity values of 0.39–0.44. *P. florida* had significant differences (*p* < 0.05) in colour analysis, with the highest lightness (L* = 75.45) and colour saturation. *P. florida* had the highest total phenolic content (58.91 mg GAE/g) and antioxidant activity (ABTS and DPPH: 42.21 and 46.12 µmol TE/g). Of the three species, *P. florida* had the highest total phenol profile, at 44.10 mg/g. Gallic acid was the predominant phenol with the highest concentration of 12.82 mg/g. The study concluded that all three common mushroom species, *P. ostreatus*, *P. florida*, and *P. pulmonarius*, are rich in nutrients, with *P. florida* standing out in bioactive compounds.

## 1. Introduction

The therapeutic properties and nutritional value of mushrooms have been studied worldwide. Unlike many other foods categorised as functional foods, mushrooms contain macronutrients such as proteins and carbohydrates, dietary fibre, and an extensive range of bioactive compounds that have positive health effects [1]. These bioactive compounds, in particular flavonoids, phenolic compounds, vitamins, and polysaccharides, contribute significantly to the antioxidant activity. Antioxidants and antioxidant activity play vital roles in modulating oxidative stress and the chronic diseases associated with it [2].

Natural antioxidants have recently been in high demand, prompting a search for alternative, naturally sourced alternatives to synthetic counterparts, which pose various health risks [3]. Among the many fungi-derived antioxidants, mushrooms, particularly those grown in controlled environments, have been shown to provide a reliable source of naturally occurring antioxidants. The many factors associated with mushroom cultivation, as well as the mushrooms themselves, alter the naturally occurring antioxidants in the mushrooms [4]. In Nigeria, the cultivation of mushrooms is a new and thriving business that is helping the country to feed its population more stably and providing an income and new nutrients [5]. The cultivation of many different species of edible mushrooms is made possible by the great climate of Ile-Ife in Osun State. Although researchers have reported on the nutritional quality of Pleurotus species, there is limited information on the geographical distribution and phenolic profiles of the three Pleurotus species studied [4,5]. The cultivation of mushrooms in the area is a new activity, and the nutritional value and antioxidant activity of the different varieties have yet to be compared [6].

Evaluating properties such as physicochemical properties (pH, TTA, TSS, and water activity), proximate composition (moisture content, ash, crude protein, lipid, and carbohydrate), total phenolic content, antioxidant activity, and phenolic profile contribute to the understanding of the nutritional quality and functional potential of these mushrooms. This type of analysis helps consumers make informed decisions, and it aids in formulating functional mushroom-based products and nutraceuticals. Thus, the objective of this study is to compare the physicochemical composition, antioxidant properties, and phenolic profile of three edible mushroom varieties grown in Ile-Ife, Osun State, Nigeria. The study aims to explain the variations among the species in question, thereby enhancing knowledge of locally cultivated mushrooms, their potential health benefits, and their contribution to sustainable food systems.

## 2. Materials and Methods

### 2.1. Materials

The edible mushrooms (*Pleurotus ostreatus*, *Pleurotus florida*, and *Pleurotus pulmonarius*) were purchased from local farmers in three different batches (i.e., three independent batches) and identified at the Department of Botany, Obafemi Awolowo University, Ile-Ife. The batches were treated separately for analysis. The mushrooms were prepared at the Department of Food Technology’s pilot plant at the Federal Polytechnic Offa, Kwara State, Nigeria. The chemicals for the analysis were purchased from Merck, South Africa, and they are all of analytical grade.

### 2.2. Sample Preparation

The mushrooms were washed with distilled water, cut into smaller pieces with a knife, and dried in a hot-air oven at 60 °C for 24 h. The dried mushrooms were milled into flour using a Vitamix 5200 (Blendtec, Durban, Kwazulu-Natal, South Africa), packed in a zip-lock bag, and stored in the refrigerator for further analysis.

### 2.3. Proximate Composition of the Three Common Edible Mushrooms

The AOAC [7] method was used to measure the moisture, ash, protein, fat, and crude fibre contents of the mushroom flour samples. Using the formula carbohydrates (%) = 100 − (moisture + ash + fat + protein + crude fibre), the carbohydrate content was determined as the difference between the percentages of moisture, fat, protein, ash, and crude fibre.

### 2.4. Determination of pH, Total Soluble Solids (°Brix), Water Activity (aW), and Total Titratable Acidity (TTA) of the Three Common Edible Mushrooms

The pH value of the mushroom flour samples was determined according to the AOAC [7] official method with slight modifications. Briefly, 10 g of mushroom flour was dispersed in 100 mL of distilled water (1:10, *w*/*v*) and homogenised using a magnetic stirrer for 5 min. The suspension was allowed to equilibrate at 25 °C for 30 min, with intermittent stirring to ensure complete hydration of the sample.

A digital pH meter (Hanna Instrument, Poroa de Varzim, Portugal) equipped with a glass electrode was calibrated before use with standard buffer solutions of pH 4 and 7. The electrode was rinsed thoroughly with distilled water and gently wiped dry between measurements. The electrode was immersed directly into the sample suspension, and the pH was recorded once the reading stabilised. Measurements were performed in triplicate, and the results were expressed as mean ± standard deviation (SD).

A handheld refractometer (Hanna Instruments, Villafranca Padovana, Italy) that had been zeroed with distilled water was used to measure total soluble solids in Brix. After each analysis, distilled water was used to clean the refractometer prism. The AOAC [7] method was used to determine the titratable acidity of the samples, and a water activity meter was used to quantify the water activity.

### 2.5. Mineral Analysis of the Three Common Edible Mushrooms

ICP emission spectroscopy (ICP-AES, Jarrel-Ash, Berkhamsted, Hertfordshire, UK) was used to measure the mineral content of the mushroom flour samples using the method outlined by Mudau et al. [8]. A muffle furnace was used to burn 2 g of the mushroom flour samples to white ash for 3 h at 550 °C. White ash was then mixed with 5 mL of nitric acid and 10 mL of hydrochloric acid solution. After 1 h in a water bath, 10 millilitres of hydrochloric acid were added, and the mixture was diluted to 100 millilitres in a 100-millilitre volumetric flask. Distilled water was added to a volumetric flask to reach a volume of 100 mL. The flour sample was analysed by ICP-AES for mineral analysis, and the results were reported as mg per 100 g.

### 2.6. Total Phenol (TPC) Determination of the Three Common Edible Mushrooms

The mushroom flour samples (50 g) were suspended in 500 mL of methanol to create the extract. After two hours of shaking, the extract was centrifuged for twenty minutes at 3000 rpm using a Rotina 380 R-Labotec Ecotherm (Pty) Ltd., Midrand, South Africa. Before use, the extract was collected, filtered through Whatman No. 1 paper, and stored at 4 °C after drying for 12 h at 45 °C.

According to Mahloko et al. [9], the modified Folin–Ciocalteu method was used to calculate the TPC of the mushroom flour samples. In a nutshell, 2 mg of dried mushroom flour extract was suspended in 1.5 mL of Folin–Ciocalteu reagent (diluted 1:10 with distilled water) and left to rest at room temperature for 5 min. A 7% sodium bicarbonate solution (2.5 mL) was then added. After one minute of vortexing, the supernatant was centrifuged for ten minutes at 5000 rpm. A UV spectrophotometer (VIS-7220N, Rayleigh Analytical Instrument Co., Ltd., Beijing, China) with a wavelength of 765 nm was used to measure the absorbance of flour samples.

### 2.7. Antioxidant Activity (ABTS, DPPH and FRAP) of the Mushroom Samples

The technique outlined by Awika et al. [10] was used to assess the ABTS radical scavenging activity of mushroom flour samples. A total of 58 mL of phosphate-buffered saline (pH 6.9) was mixed with exactly 2 mL of ABTS stock solution (0.01 M), then left in the dark for 12 h. The reaction mixture was incubated for 30 min after 10 μL of the sample was combined with 190 μL of ABTS. Trolox was used as the standard to quantify the absorbance at 734 nm. The results were reported as micromoles of Trolox equivalents per milligram of material (μmol TE/mg).

The DPPH assay of the mushroom flour samples was measured using a slightly modified version of the procedure described by Ramatsetse et al. [11]. A total of 1.465 mg was dissolved in 50 mL of methanol to create the DPPH solution. Two millilitres of extract from mushroom flour samples were suspended in methanol. The mixture was then diluted to 25 mL with methanol and 1 mL of DPPH solution was added. The supernatant was well agitated at room temperature (25 ± 2 °C) and then incubated in a dark room for 30 min. The absorbance at 570 nm was measured using an ultraviolet spectrophotometer (Beckman Coulter, South Pasadena, CA, USA) and the results are expressed as a percentage of DPPH.

With a few minor adjustments, the FRAP of the mushroom flour samples was analysed as described by Mudau and Adebo [12]. In short, 300 mL of distilled water was added to a test tube containing 100 µL of the mushroom flour sample. Next, 250 µL of the mushroom sample flour was mixed with 3 mL of FRAP reagent. After vortexing, the mixture was left in a water bath at room temperature for 20 min. The absorbance of the sample at 593 nm was measured using a UV-Vis spectrophotometer (Beckman Coulter, South Pasadena, CA, USA); results are expressed as µmol Fe^2+^/g.

### 2.8. Colour Determination

The colour characteristics (L*, a*, and b*) of the mushroom flour samples were analysed using a colourimeter (Hunter Lab ColorFlex, Reston, VA, USA) in compliance with Mudau et al. [8]. The device was calibrated using a white and black plate, which is an industry standard. Hunter values for L* (lightness), a* (redness), and b* (yellowness) were used to express colour readings. On the extracted MRFJ, three measurements were made directly. Equations (1) and (2) were utilised to determine Chroma (C) and Colour Change (ΔE) based on the colour attributes (L*, a*, b*):

(1) $$Chroma,\;\left(C\right)=\sqrt{a^{2}+b^{2}}$$

(2) $$Total\;colour\;difference\left(\Delta E\right)=\sqrt{{\left(\Delta L*\right)}^{2}+{\left(\Delta a*\right)}^{2}+(\Delta {b*)}^{2}}$$

### 2.9. Determination of Phenolic Acids

#### 2.9.1. Sample Preparation

100 mg of the mushroom flour sample was placed into a test tube containing 1 mL of 70% methanol. After vortexing the supernatant, it was incubated for 180 min at 60 °C. A 130 µL aliquot of the extract was derivatised with 30 µL BSTFA and 100 µL acetonitrile for 30 min at 60 °C after being dried overnight in a freeze dryer. The flour sample was then placed in an insert (a 2 mL GC vial), and 1 µL was injected into the GC-MS/MS in splitless mode.

#### 2.9.2. Chromatographic Separation

The phenolic acids were analysed using a triple quadrupole mass spectrometer (MS 7697A, Agilent Technology, Shanghai, China) connected to a gas chromatograph (GC 7820A, Agilent Technology, Shanghai, China). In short, a nonpolar Rxi-5Sil MS capillary column (30 m, 0.25 mm ID, 0.25 µm film thickness) was used to separate the analytes. At a flow rate of one millilitre per minute, helium was employed as the carrier gas. The injector temperature was maintained at 250 °C. Splitless mode was used to inject one microliter of the sample. The oven temperature was then adjusted as follows: 100 °C for 4 min, then 180 °C at 10 °C/min for 2 min, and finally 320 °C at 20 °C/min for 5 min. The source and quad temperatures were kept at 250 °C and 150 °C, respectively, while the mass spectrometer detector operated in a single reaction mode. The transfer line temperature was maintained at 250 °C. Calibration curves for gallic acid, catechin, chlorogenic acid, caffeic acid, p-coumaric acid, ferulic acid, quercetin, and kaempferol were constructed using eight different concentrations (1–100 µg/mL). Perfect linearity was obtained for all the analytes, with coefficients of determination (R^2^) ranging from 0.9992 to 0.9997. The limits of detection (LOD) ranged from 0.08 to 0.15 µg/mL, while the limits of quantification (LOQ) ranged from 0.27 to 0.48 µg/mL.

### 2.10. Statistical Analysis

Results from the mushroom flour samples were statistically analysed using IBM SPSS Version 25. The mean and standard deviation of the mushroom flour samples were reported in triplicate. To account for natural variability, homogeneity analyses were performed on mushroom samples. One-way ANOVA was used to assess the results. To find significant differences between mean values (*p* < 0.05), the Least Significant Difference (LSD) test was used.

## 3. Results and Discussions

### 3.1. Proximate Composition of the Edible Mushrooms

Table 1 shows the proximate analysis of the three commercially grown edible mushrooms—Oyster mushrooms (Pleurotus ostreatus), *P. florida* and *P. pulmonarius*—grown in Ile-Ife, Nigeria. The moisture content of the mushrooms was between 9.56% in *P. florida* and 11.01% in *P. pulmonarius*. The *P. pulmonarius* contained the highest moisture content, whereas *P. ostreatus* contained 10.14% moisture, and *P. florida* contained the least moisture content (9.56%). These values are within the previously reported range for Pleurotus species (8–12%), indicating that Pleurotus species exhibit good drying efficiency [13,14]. The lower moisture content in *P. florida* compared to the other two species may indicate *P. florida* has better storage ability and is less vulnerable to microbial spoilage.

*P. ostreatus* contains the highest amount of crude protein (26.12%). *P. pulmonarius* contained crude protein (25.59%), and *P. Florida* contained the lowest amount of crude protein (24.21%). Compared with other Pleurotus species, these values are noteworthy and confirm that Pleurotus species are highly recommended for protein sourcing (high-quality fungal protein) [15]. Differences in these proximate composition parameters can be attributed to genetic variability, growth substrates, or environmental factors that may differ in Ile Ife. The content of crude fat is very low for all the mushrooms (3.45–3.70%), and there is no statistically significant difference (*p* > 0.05). The lipid content of Pleurotus mushrooms is very low, which supports the idea that mushrooms can be part of low-fat functional foods [16].

The ash content, which serves as a measure of total mineral matter, ranged from 7.24 ± 0.22% (*P. florida*) to 8.13 ± 0.12% (*P. pulmonarius*). These relatively high ash values indicate a rich mineral composition (especially potassium, phosphorus, magnesium, and other trace minerals) in these mushroom species, which are known to take up minerals from their growing substrate [17]. There was higher ash content found in *P. pulmonarius* and P. ostreatus, compared to *P. florida*, thus indicating greater potential for mineral bioaccumulation. *P. ostreatus* recorded the highest value (9.77%) for crude fibre, while *P. florida* recorded the lowest crude fibre (8.91%). These values are consistent with the previously reported range (7–12%) and demonstrate that these mushrooms can contribute significantly to dietary fibre, which improves digestive health and glycemic control [18].

The carbohydrate content (calculated by difference) ranged from 42.17% (*P. pulmonarius*) to 46.38% (*P. florida*). The carbohydrate content is also influenced by the presence of chitin, β-glucans, and other polysaccharides that contribute to the mushrooms’ dietary fibre and other bioactive properties. It is therefore valid to suggest that all three species of the genus Pleurotus have a high protein content (24–26% range), moderate fibre content (9–10% range), and low-fat content, indicating that they can serve as functional, nutrient-dense food products. *P. ostreatus* had the highest protein and fibre content, while *P. Florida* had the lowest moisture content and the highest carbohydrate content. These findings justify promoting locally grown oyster mushrooms in Nigeria as a cheap, high-quality protein source, especially to combat protein-energy malnutrition and micronutrient deficiencies.

### 3.2. Physicochemical Properties of the Three Common Edible Mushrooms

Table 2 summarises the physicochemical characteristics of three popular edible mushrooms currently commercially cultivated in Ile-Ife, Nigeria. Such parameters are pH, titratable acidity, water activity, total soluble solids, etc. These are key factors that influence microbial stability, sensory quality, and postharvest shelf life of mushrooms. The pH values are close, ranging between 6.12 for *P. florida* and 6.31 for *P. ostreatus*. There are no statistically significant differences in pH between the species (*p* > 0.05). This near-neutral pH (6.0–6.8) is common among members of the genus *Pleurotus*. Bacteria and moulds that cause food spoilage can grow in environments with pH values above 5.5. The high pH of these mushrooms makes them highly perishable; postharvest, they should be cooled immediately or processed to maintain quality.

For all samples, titratable acidity (TTA) was low, ranging from 0.22–0.26%, and there were no significant differences. This matches the low acidity and the citric, malic, and succinic acid profiles of oyster mushrooms [19]. Low acidity may also reduce microbial spoilage; without proper raw chain management and preservation techniques, it may increase the product’s exposure to spoilage. The Water activity (aW) values range from 0.39 (*P. florida*) to 0.44 (*P. pulmonarius*). Among the evaluated mushroom flour samples, *P. florida* showed the lowest water $a_{w}$ value (0.39), followed by *P. ostreatus* (0.42), while *P. pulmonarius* had the highest value (0.44). The statistical differences among the mushroom flour samples (*p* < 0.05) suggest species-specific differences in drying behaviour, cellular composition, and water-retention properties [20]. The lower $a_{w}$ value obtained for *P. florida* indicates greater dehydration efficiency and potentially improved storage stability compared with the other mushroom flours.

Total soluble solids (TTS, °Brix) ranged from 3.61 (P. ostreatus) to 4.11 (*P. florida*) without any significance amongst species (*p* > 0.05). Low TTS is associated with high moisture and low free sugar concentration, which are characteristic of fresh Pleurotus mushrooms. Values of TTS for Pleurotus species in this study are comparable to those reported for tropical oyster mushrooms (3.0–5.0 °Brix) [21]. Changes in TTS for *P. florida* indicate possible differences in soluble sugar concentration, which could, to some extent, affect mushroom sweetness and their potential for value-added product development. The three *Pleurotus* species exhibited broadly similar physicochemical profiles, with minor but consistent differences likely attributable to genetic variation, substrate composition, or micro-environmental conditions during cultivation in Ile-Ife.

The combination of near-neutral pH, low titratable acidity, and high-water activity confirms the perishability of these mushrooms and demonstrates the need for improved postharvest handling, cold storage, or processing technologies (drying, canning and fermentation) to address the significant postharvest losses (often >40%) reported for mushrooms in Nigeria [22].

### 3.3. Mineral Composition of Three Common Edible Mushrooms

Table 3 (mg/100 g, dry weight basis) shows the mineral analysis of the three most popular edible mushrooms, collected from Ile-Ife, Nigeria, which are *Pleurotus ostreatus*, *P. florida,* and *P. pulmonarius*. These mushrooms are rich in minerals, and the distribution of elements in the mushrooms is as follows: potassium (K) > phosphorus (P) > calcium (Ca) > magnesium (Mg) > iron (Fe) > zinc (Zn). *P. Pulmonarius* and *P. florida* scored the highest of all the samples with potassium concentrations that ranged from 1673 mg/100 g (*P. florida*) to 1855 mg/100 g (*P. pulmonarius*). *P. Pulmonarius* and *P. ostreatus* have significantly higher levels of potassium than *P. florida*. These levels are considered extremely high, and it is well established that mushrooms are a rich source of electrolytes, which are vital for regulating blood pressure and supporting muscle function [16].

Calcium (Ca) concentration also varied among the three samples, with the highest value recorded for *P. florida* (38.76 mg/100 g), followed by *P. pulmonarius* (35.33 mg/100 g) and P. ostreatus (32.18 mg/100 g).

Although the calcium concentrations in these samples were not as high as those of potassium, they are considered significant and contribute greatly as a source of calcium and as a food source in general, especially within the context of subsistence populations with little to no access to dairy. Magnesium (Mg) concentration was also the highest in *P. ostreatus* (18.11 mg/100 g), followed by *P. pulmonarius* (16.15 mg/100 g) and finally *P. florida* (11.22 mg/100 g). Magnesium is a cofactor in many enzyme systems and is also essential in energy metabolism, and the concentrations of magnesium in these mushrooms are comparable to other sources of leafy vegetables [23].

Iron (Fe) content ranged from 15.71 mg/100 g (*P. florida*) to 16.32 mg/100 g (*P. ostreatus*). These values are relatively high for plant-based foods and suggest strong potential to combat iron-deficiency anaemia, a prevalent nutritional disorder in Nigeria [24]. Phosphorus (P) followed a similar pattern to potassium, with *P. ostreatus* and *P. pulmonarius* showing significantly higher levels (312.41 and 305.23 mg/100 g, respectively) than *P. florida* (289.11 mg/100 g). The Ca:P ratios across the species (approximately 1:9 to 1:8) are within the optimal range for human nutrition and bone health when incorporated in a balanced diet [25]. Of the species analysed, *P. ostreatus* had the highest levels of Zinc (Zn) at 4.12 ± 0.25 mg/100 g, and *P. florida* recorded the lowest levels of Zn at 3.61 mg/100 g. Zn is important for immunity and growth, and Zn levels in the three species are comparable to those in other edible mushrooms, making the Pleurotus species important from a vegetarian diet perspective [26]. The three species of Pleurotus mushroom analysed are important sources of essential, limited minerals, particularly potassium and phosphorus, and have moderate to high levels of calcium, magnesium, iron and zinc. *P. ostreatus* recorded the better mineral composition and was followed closely by *P. pulmonarius*, while *P. florida* recorded lower values for most minerals considered in the studies.

### 3.4. Total Phenolic Content and Antioxidant Activities of Three Common Edible Mushrooms

Table 4 shows total phenolic content (TPC) and antioxidant activities (ABTS, DPPH, and FRAP) of three popular edible mushrooms, Pleurotus ostreatus, *P. florida*, and *P. pulmonarius* from Ile-Ife, Nigeria. Total Phenolic Content (TPC) was 58.91 mg GAE/g (*P. florida*) and 42.13 mg GAE/g (*P. pulmonarius*). Mean *P. ostreatus* was 46.23 mg GAE/g. *P. florida* had lower TPC than the other two species (*p* < 0.05). Values of *P. ostreatus*, *P. pulmonarius*, and *P. florida* correspond to reported phenolic content, which reflects significant phenolic load, due to phenolic acids and flavonoids [27]. Factors contributing to lower TPC in *P. pulmonarius* are likely genetic or cultivation substrate-related, as phenolic accumulation is strain-dependent in oyster mushrooms [28].

Of the three species analysed (all with *p* < 0.05), *P. florida* had the highest ABTS scavenging activity (42.21 µmol TE/g), followed by *P. ostreatus* (39.72 µmol TE/g) and *P. pulmonarius* (35.67 µmol TE/g), with all species being significantly different. Although the three species were all confirmed to have antioxidant capacity by DPPH and FRAP assays, *P. florids* showed the highest antioxidant capacity. Previous studies observed a strong, positive relationship (>0.85) between TPC and various antioxidant activities (ABTS and DPPH), demonstrating that phenolics are likely the compounds with the greatest potential to reduce free radical scavenging in mushrooms [29,30]. The discrepancy between *P. pulmonarius* and the other species is also noteworthy, demonstrating that *P. florida* and *P. ostreatus* have the highest antioxidant activity, which can be used to produce functional foods and nutraceuticals.

Ferric Reducing Antioxidant Power (FRAP) analysis showed *P. florida* and *P. ostreatus* had higher reducing ability. Both species showed FRAP values of 79.23 and 75.44 µmol Fe^2+^/g, respectively, compared to *P. pulmonarius* at 67.33 µmol Fe^2+^/g. The FRAP assay is known for its ability to evaluate the electron-donating power of antioxidants, estimate the presence of reductive phenolic compounds, and assess the ability of antioxidants to convert Fe^3+^ to Fe^2+^ in acidic conditions. The comparatively lower FRAP value in *P. pulmonarius* indicated lower reducing power and potentially lower levels of phytochemicals that donate electrons. These outcomes were consistent with studies showing that the antioxidant activity of mushrooms varies with species, substrate composition, cultivation conditions, and genetic factors [28].

*P. florida* had the greatest potential to serve as a natural source of antioxidant compounds for both the pharmaceutical and nutraceutical sectors, as well as a functional food ingredient. Nonetheless, the lower levels of antioxidant activity in *P. ostreatus* and *P. pulmonarius* still warrant consideration for dietary inclusion, given the known health benefits of edible mushrooms. Supplementary Figure S1 shows a correlation between TPC and all the antioxidant properties of the mushrooms. There is a positive correlation between TPC and ABST (0.9965), DPPH (0.9972), and FRAP (0.9958). Given this positive correlation, it could be said that TPC was responsible for the mushrooms’ antioxidant activity.

### 3.5. Colour Characteristics of Three Common Edible Mushrooms

The colour of fresh mushrooms is associated with quality and significantly affects customer acceptance, as they have a higher market value and greater nutritional value than older mushrooms. The colour parameters of Pleurotus species cultured in Ile-Ife, as shown in Table 5 (L*: lightness, a*: red/green; b*: yellow/blue; C*: chroma; ΔE*: total colour difference), give a quantitative measure of the colour differences attributed to the species.

There is substantial evidence of significant (*p* < 0.05) differences in colour attributes between the three varieties of mushrooms. *P. florida* contains the highest value of lightness with a value of 75.45, while *P. ostreatus* and *P. pulmonarius* contain 72.15 and 70.23, respectively. Consumers tend to prefer higher levels of lightness because it conveys a fresher, higher-quality product. *P. pulmonarius* has the lowest L* value; hence it was darker, which could reflect differences in the level of pigmentation, enzymatic browning, or a difference in the phenolic content as compared to the other species.

*P. pulmonarius* has the highest value (2.04) of a*, while *P. Ostreatus* was 1.85, and *P. florida* has the lowest with 1.43. This shows a small shift towards red, which may represent differences in pigmentation between the mushrooms or in their tissues during the post-harvest stage. In previous research, an increase in the value of a* for mushrooms was attributed to increased enzymatic browning reactions resulting from higher levels of polyphenol oxidase [31]. For b* (yellowness), *P. Florida* has the highest with 13.11, followed by *P. ostreatus* with 12.34 and *P. pulmonarius* with 11.79. This trend is reflected in chroma (C*), which measures colour saturation. The b* and C* values in *P. florida* indicate it is the most saturated and vivid in colour and thus the most visually appealing. The colour saturation of mushrooms is due to the presence of carotenoids and other pigments, moisture content, and surface characteristics [32].

The total colour difference (ΔE*) also indicates the level of visual distinction among the samples. *P. ostreatus* is the reference with ΔE* = 0.00 as expected. *P. florida* has the highest ΔE* at 3.68 and is the most distinguished in colour. *P. pulmonarius* has a ΔE* of 2.12, which is moderate. ΔE* values greater than 2–3 are detectable by the human eye [33]. In this case, the colour differences among the species are great and are likely to affect consumer preferences. The differences in colour characteristics among the three Pleurotus species may result from a combination of internal and external factors. In this case, internal factors refer to genetic differences, while external factors include substrate composition, different environmental conditions, and handling conditions. Different characteristics have also been documented in cultivated mushrooms. This shows that species-specific characteristics are essential in defining the visual quality [34].

### 3.6. Phenolic Profile (mg/100 g Dry Weight) of Three Common Edible Mushrooms

The phenolic constituents of the three species of Pleurotus shown in Table 6 show significant (*p* < 0.05) interspecific differences both in individual phenolic compounds and in total phenolic content (TPC). *P. florida* had the highest values in all the quantified phenolic compounds, while *P. pulmonarius* had the lowest across individual and total phenolic content. With regard to total phenolic content, *P. florida* (44.10 mg/100 g) had a concentration greater than P. *ostreatus* (36.04 mg/100 g) and *P. pulmonarius* (30.56 mg/100 g).

Among the different species, gallic acid was the dominant compound, ranging from 8.91 to 12.82 mg/100 g. This agrees with previous findings that gallic acid is the most abundant phenolic compound in the Pleurotus species and exhibits antioxidant activity [35].

The highest level recorded in *P. florida* indicates greater biosynthetic production of hydroxybenzoic acid derivatives. Catechin and, especially, chlorogenic acid were also recorded at commendable levels, with *P. florida* once again leading with 7.65 and 6.92 mg/100 g, respectively. These compounds are also known for their strong radical-scavenging potential, thus contributing to the overall antioxidant activity of the fungi. The presence of phenolic acids and flavonoids classifies the Pleurotus species as functional foods with potential health benefits [36].

Similarly, *P. florida* recorded the highest levels of the hydroxycinnamic acids, caffeic, p-coumaric and ferulic acids, with ferulic acid being the most abundant in this category (5.34 mg/100 g in *P. florida*). These phenolic acids are associated with oxidation, inflammation, and microbial infection, and their presence in mushrooms is ascribed to lignocellulosic structure and metabolism [37]. The presence of flavonoids such as quercetin and kaempferol indicates lower levels of phenolic acids than of flavonoids, which is consistent with the findings of most researchers. The contribution of lower levels of flavonoids is to enhance antioxidant activity, particularly free radical scavenging and metal ion chelation [38].

The differences among the three Pleurotus species are attributed to many factors, including genetics, environmental conditions, and substrate composition. As secondary metabolites, Pleurotus species phenolics are added/synthesized to/degraded lignocellulosic materials [34]. In addition, the greater accumulation of phenolics in *P. florida* can be attributed to the differences in the activities of enzymes involved in the phenylpropanoid pathway. The range of phenolic compounds recorded in the current study for edible mushrooms (1–6 mg/g DW = 100–600 mg/100 g) is comparable to that in the *P. florida* phenolic profile, excluding the differences in extraction methods and units [39].

The findings provide evidence for selecting other *P. florida* species for cultivation under the climatic conditions of Ile-Ife and other regions. The *P. florida* species is rich in phenolic compounds and has strong potential for use in functional foods and other nutraceutical applications. The results also support the selection of species for mushroom cultivation in Ile-Ife, as the local climate can influence the phytochemical profile of the mushrooms.

## 4. Conclusions

This research outlines the nutritional value of mushrooms in Ile-Ife, Nigeria. All three Pleurotus species demonstrated high nutritional and functional value, with high protein, moderate fibre, low fat, and a high mineral composition. *P. ostreatus* had the highest protein and minerals, *P. florida* had the highest antioxidant activity, and *P. pulmonarius* had the lowest. Among the three Pleurotus species, *P. florida* is the best because of its higher total phenolic content and phenolic profile. Consumption of *P. florida* may improve human health. Their use, particularly in Nigeria, would help address the country’s nutritional problem.

## Figures and Tables

**Table 1:** Proximate composition of three common edible mushrooms (*P. ostreatus*, *P. florida & P. pulmonarius*) common in Ile-Ife, Nigeria (% DWB).

Samples	Moisture	Crude Protein	Crude Fat	Ash	Crude Fibre	Carbohydrate
*P. ostreatus*	10.14 ± 0.12 ^b^	26.12 ± 0.12 ^b^	3.45 ± 0.52 ^a^	7.81 ± 0.12 ^b^	9.77 ± 0.12 ^b^	42.71 ± 0.67 ^b^
*P. florida*	9.56 ± 0.20 ^a^	24.21 ± 0.22 ^a^	3.70 ± 0.42 ^a^	7.24 ± 0.22 ^a^	8.91 ± 0.15 ^a^	46.38 ± 0.55 ^c^
*P. pulmonarius*	11.01 ± 0.14 ^c^	25.59 ± 0.30 ^b^	3.55 ± 0.20 ^a^	8.13 ± 0.12 ^c^	9.55 ± 0.22 ^b^	42.17 ± 0.42 ^a^

The values are mean ± SD of triplicate determinations. Values followed by the different superscript(s) within the column differ significantly (*p* ≤ 0.05). The Least Significant Difference (LSD) test was performed to identify significant differences between mean values (*p* < 0.05).

**Table 2:** Physicochemical properties of three common edible mushrooms (*P. ostreatus*, *P. florida & P. pulmonarius*) common in Ile-Ife, Nigeria.

Samples	pH	TTA (%)	Water Activity (aW)	TTS (°Brix)
*P. ostreatus*	6.31 ± 0.20 ^a^	0.23 ± 0.02 ^a^	0.42 ± 0.02 ^a^	3.61 ± 0.10 ^a^
*P. florida*	6.12 ± 0.10 ^a^	0.26 ± 0.01 ^a^	0.39 ± 0.01 ^a^	4.11 ± 0.11 ^b^
*P. pulmonarius*	6.24 ± 0.12 ^a^	0.22 ± 0.02 ^a^	0.44 ± 0.02 ^a^	3.72 ± 0.32 ^a^

The values are mean ± SD of triplicate determinations. Values followed by the different superscript(s) within the column differ significantly (*p*  ≤  0.05). The Least Significant Difference (LSD) test was performed to identify significant differences between mean values (*p* < 0.05).

**Table 3:** Mineral composition of three common edible mushrooms (*P. ostreatus*, *P. florida & P. pulmonarius*) common in Ile-Ife, Nigeria (mg/100 g).

Samples	K	Ca	Mg	Fe	P	Zn
*P. ostreatus*	1817 ± 2.02 ^b^	32.18 ± 0.31 ^a^	18.11 ± 0.30 ^c^	16.32 ± 0.22 ^b^	312.41 ± 0.50 ^b^	4.12 ± 0.25 ^b^
*P. florida*	1673 ± 1.12 ^a^	38.76 ± 0.35 ^c^	11.22 ± 0.42 ^a^	15.71 ± 0.12 ^a^	289.11 ± 0.72 ^a^	3.61 ± 0.12 ^a^
*P. pulmonarius*	1855 ± 1.50 ^b^	35.33 ± 0.52 ^b^	16.15 ± 0.22 ^b^	16.11 ± 0.32 ^b^	305.23 ± 0.92 ^b^	3.92 ± 0.22 ^a^

The values are mean ± SD of triplicate determinations. Values followed by the different superscript(s) within the column differ significantly (*p*  ≤  0.05). The Least Significant Difference (LSD) test was performed to identify significant differences between mean values (*p* < 0.05).

**Table 4:** Total phenolic content and antioxidant activities of three common edible mushrooms (*P. ostreatus*, *P. florida & P. pulmonarius*) common in Ile-Ife, Nigeria.

Samples	TPC (mg GAE/g).	ABTS (µmol TE/g)	DPPH (µmol TE/g)	FRAP (µmol Fe^2+^/g)
*P. ostreatus*	46.23 ± 0.42 ^b^	39.12 ± 0.62 ^b^	30.71 ± 0.34 ^b^	75.44 ± 0.52 ^b^
*P. florida*	58.91 ± 0.35 ^c^	42.21 ± 0.31 ^c^	46.12 ± 0.30 ^c^	79.23 ± 0.30 ^c^
*P. pulmonarius*	42.13 ± 0.31 ^a^	35.67 ± 0.35 ^a^	29.22 ± 0.25 ^a^	67.33 ± 0.52 ^a^

The values are mean ± SD of triplicate determinations. Values followed by different superscript(s) within the column differ significantly (*p*  ≤  0.05). The Least Significant Difference (LSD) test was performed to identify significant differences between mean values (*p* < 0.05).

**Table 5:** Colour characteristics of three common edible mushrooms (*P. ostreatus*, *P. florida & P. pulmonarius*) common in Ile-Ife, Nigeria.

Samples	L*	a*	b*	C*	ΔE*
*P. ostreatus*	72.15 ± 0.32 ^b^	1.85 ± 0.32 ^b^	12.34 ± 0.32 ^b^	12.48 ± 0.12 ^b^	0.00 ± 0.00 ^a^
*P. florida*	75.45 ± 0.32 ^c^	1.43 ± 0.32 ^a^	13.11 ± 0.32 ^c^	13.19 ± 0.30 ^c^	3.68 ± 0.12 ^c^
*P. pulmonarius*	70.23 ± 0.32 ^a^	2.04 ± 0.32 ^c^	11.79 ± 0.32 ^a^	11.96 ± 0.22 ^a^	2.12 ± 0.15 ^b^

The values are mean ± SD of triplicate determinations. Values followed by different superscript(s) within the column differ significantly (*p*  ≤  0.05). The Least Significant Difference (LSD) test was performed to identify significant differences between mean values (*p* < 0.05).

**Table 6:** Phenolic profile of three common edible mushrooms (*P. ostreatus*, *P. florida* & *P. pulmonarius*) common in Ile-Ife, Nigeria.

Phenolic Compounds	*P. ostreatus*	*P. florida*	*P. pulmonarius*
Gallic acid	10.45 ± 0.40 ^b^	12.82 ± 0.60 ^c^	8.91 ± 0.25 ^a^
Catechin	6.73 ± 0.30 ^b^	7.65 ± 0.20 ^c^	5.84 ± 0.30 ^a^
Chlorogenic acid	5.58 ± 0.12 ^b^	6.92 ± 0.15 ^c^	4.96 ± 0.31 ^a^
Caffeic acid	3.21 ± 0.10 ^b^	4.03 ± 0.20 ^c^	2.87 ± 0.15 ^a^
p-Coumaric acid	3.15 ± 0.11 ^b^	3.68 ± 0.20 ^c^	2.22 ± 0.13 ^a^
Ferulic acid	4.12 ± 0.25 ^b^	5.34 ± 0.12 ^c^	3.68 ± 0.15 ^a^
Quercetin	1.84 ± 0.02 ^b^	2.41 ± 0.10 ^c^	1.36 ± 0.01 ^a^
Kaempferol	0.96 ± 0.01 ^b^	1.25 ± 0.04 ^c^	0.72 ± 0.02 ^a^
Total phenolics	36.04 ± 1.49 ^b^	44.10 ± 1.61 ^c^	30.56 ± 1.32 ^a^

The values are mean ± SD of triplicate determinations. Values followed by the different superscript(s) within the column differ significantly (*p*  ≤  0.05). The Least Significant Difference (LSD) test was performed to identify significant differences between mean values (*p* < 0.05).

## Data Availability

The original contributions presented in this study are included in the article/Supplementary Materials. Further inquiries can be directed to the corresponding author.

## Supplementary Materials

The following supporting information can be downloaded at: https://www.mdpi.com/article/10.3390/molecules31172950/s1, Figure S1: Correlation graph of TPC against ABTS, DPPH and FRAP for the different mushroom flours.
