# Supplementary material for: Comparative Analysis of Physicochemical Composition and Antioxidant Properties of Three Edible Mushroom Varieties
Source: Molecules. 2026 Aug 23;31(17):2950. doi: 10.3390/molecules31172950 (PMC13566940; doi:10.3390/molecules31172950)
Supplement: Supplementary file 1 [file molecules-31-02950-s001.zip › molecules-4474610-supplementary.pdf]

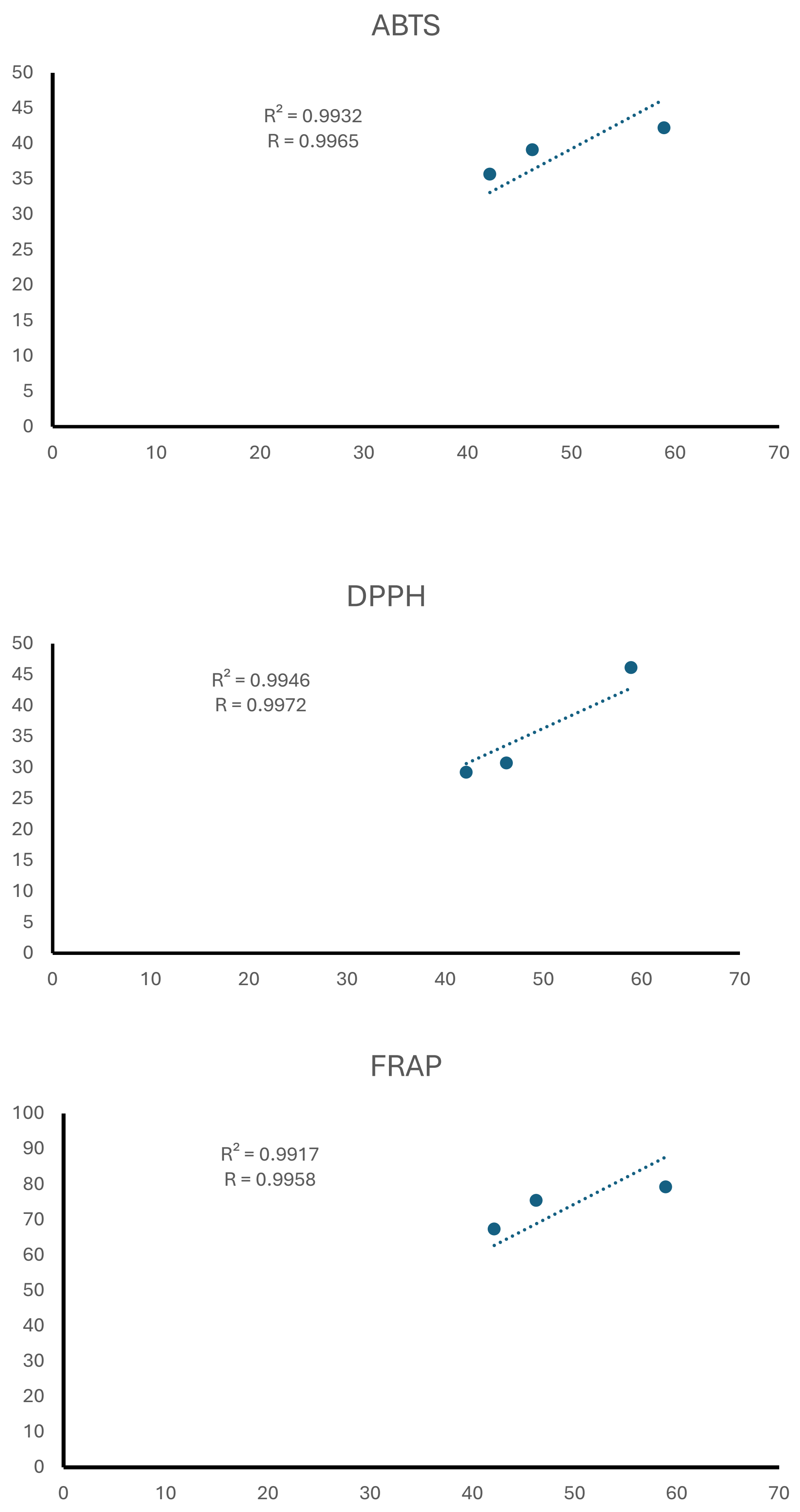

Figure S1: Correlation graph of TPC against ABTS, DPPH and FRAP for the different mushroom flours.
